# Crystal structure of a heterometallic coordination polymer: poly[di­aqua­bis­(μ_7_-benzene-1,3,5-tri­carboxyl­ato)dicalcium(II)copper(II)]

**DOI:** 10.1107/S205698901700665X

**Published:** 2017-05-12

**Authors:** Feng Zhang, Bing-Guang Zhang

**Affiliations:** a Key Laboratory of Catalysis and Materials Sciences of the State Ethnic Affairs Commission & Ministry of Education, College of Chemistry and Material Science, South-Central University for Nationalities, Wuhan 430074, People’s Republic of China

**Keywords:** crystal structure, heterometallic complex, copper carboxyl­ates, calcium carboxyl­ates, π–π stacking

## Abstract

In the title complex, [Ca_2_Cu(C_9_H_3_O_6_)_2_(H_2_O)_2_]_*n*_, the Ca^II^ and Cu^II^ cations are bridged by the benzene-1,3,5-tri­carboxyl­ate anions (BTC^3−^) to form the coordination polymer, in which each BTC^3−^ anion bridges two Cu^II^ and five Ca^II^ cations with a μ_7_ coordination mode. The Cu^II^ cation, located at an inversion centre, is in a nearly square-planar geometry defined by four O atoms from four bridging BTC^3−^ anions, while the Ca^II^ cation is in a distorted octa­hedral geometry defined by five O atoms from bridging BTC^3−^ anions and one water mol­ecule. O—H⋯O hydrogen bonds between coordinating water mol­ecules and carboxyl groups further stabilize the structure; π–π stacking is also observed between parallel benzene rings, the centroid-to-centroid distance being 3.357 (2) Å.

## Abstract 2

The CaO_6_ polyhedron and CuO_4_ quadrilateral are connected by the benzene-1,3,5-tri­carboxyl­ate anions to give a three-dimensional polymeric complex.

## Chemical context   

In recent years, the rational design and synthesis of heterometallic coordination compounds have attracted much attention due to their potential applications in magnetism, luminescence, adsorption, chemical sensing and catalysis, as well as their aesthetically beautiful architectures and topologies (Cui *et al.*, 2012 ▸; Huang *et al.*, 2013 ▸; Ma *et al.*, 2014 ▸; Wimberg *et al.*, 2012 ▸). However, hererometallic organic frame­works are investigated less frequently than single-metal organic frameworks in crystal engineering, mainly because of the competitive complexation of different metal ions in the self-assembly progress. Recently, alkaline-earth metal ions have attracted more and more research inter­est owing to their unpredictable coordination number and pH-dependent self-assembly in the construction of novel topological coordination compounds (Borah *et al.*, 2011 ▸; Chen *et al.*, 2011 ▸). However, the larger atomic radii and high enthalpy of hydration make it relatively difficult to design the coordination polymers of alkaline-earth metal ions as well as to synthesize them from aqueous solution (Reger *et al.*, 2013 ▸). As alkaline-earth metals and transition metals coordinate to the same ligand, it often gives rise to homometallic coordination compounds rather than heterometallic ones. In this regard, one of the effective synthetic strategies in building the alkaline-earth-metal-containing compounds is to employ appropriate bridging ligands. As a multifunctional hybrid ligand, H_3_BTC (benzene-1,3,5-tricarboxylic acid) in its partly or fully deprotonated form exhibits versatile coordination modes and can bind to the metal ions by making full use of the carboxyl­ate oxygen atoms. In addition, heterometallic compounds incorporating only the H_3_BTC ligand are few in number (Chen *et al.*, 2004 ▸; Li *et al.*, 2010 ▸; Sun *et al.*, 2014 ▸, 2016 ▸; Xu *et al.*, 2014 ▸). As part of our ongoing studies on these compounds, we describe here synthesis and crystal structure of the title compound, [Ca_2_Cu(BTC)_2_(H_2_O)_2_]_*n*_, (**1**).
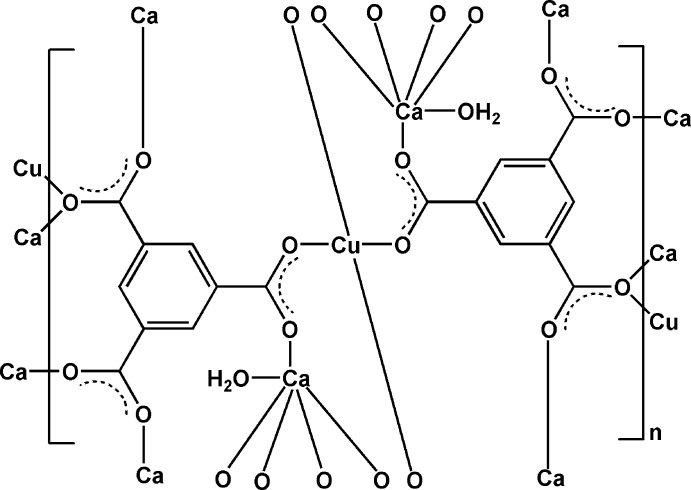


## Structural commentary   

**Figure 1:**
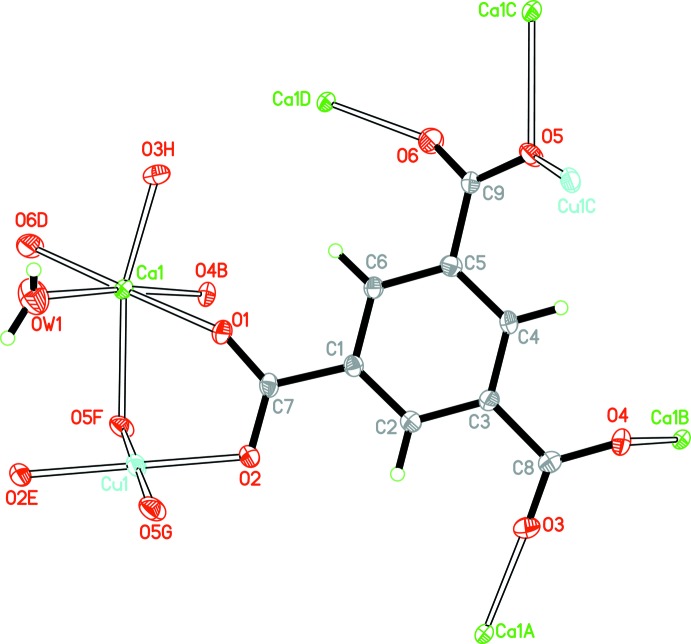
The coordination mode and atom-numbering scheme for (**1**). Displacement ellipsoids for non H-atoms are drawn at the 50% probability level, with H atoms shown as spheres of arbitrary radius. [Symmetry codes: (*A*) *x*, *y*, *z* + 1; (*B*) −*x*, −*y* + 1, −*z* + 2; (*C*) *x*, *y* − 1, *z*; (*D*) −*x*, −*y* + 1, −*z* + 1; (*E*) −*x* + 1, −*y* + 2, −*z* + 2; (*F*) *x*, *y* + 1, *z*; (*G*) −*x* + 1, −*y* + 1, −*z* + 2; (*H*) *x*, *y*, *z* − 1.]

The asymmetric unit of (**1**) contains one copper(II) cation (located at an inversion centre), one calcium(II) cation, one BTC^3−^ anion and one coordinating water mol­ecule (Fig. 1 ▸). The Cu—O bond lengths are in the range 1.9435 (19)–1.9800 (19) Å and the Ca—O bond lengths are in the range of 2.280 (2)–2.466 (2) Å (Table 1 ▸). All data are comparable to those reported for other related Cu^II^–BTC and Ca^II^–BTC complexes (Chui *et al.*, 1999 ▸; Yang *et al.*, 2004 ▸) . Each Cu^II^ cation is four-coordinated by four oxygen atoms from four different BTC^3−^ anions, forming a nearly square-planar geometry. Each Ca^II^ cation is six-coordinated by five carboxyl­ate oxygen atoms from five different BTC^3−^ anions and one terminal water mol­ecule, displaying a distorted octa­hedron (Fig. 1 ▸). The mean deviation of the equatorial plane constructed by atoms O1, O4, O6 and O*W*1 is 0.06 Å. The H_3_BTC molecule is fully deprotonated and bridges two Cu^II^ ions and five Ca^II^ ions in a μ_7_ coordination mode.

## Supra­molecular features   

**Figure 2:**
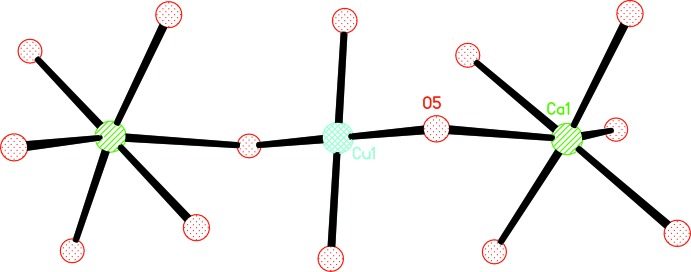
The trinuclear unit constructed from a [CaO_6_] octa­hedron and a [CuO_4_] quadrilateral.

**Figure 3:**
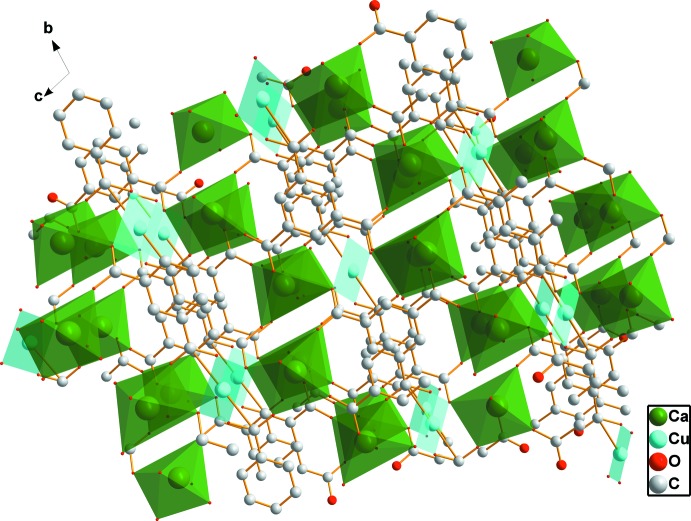
Polyhedral view of the three-dimensional heterometallic coordination framework of (**1**). All H atoms have been omitted for clarity.

Each CuO_4_ quadrilateral shares a vertex (O5) with two CaO_6_ polyhedra to form a trinuclear unit {CuCa_2_O_14_} with Ca–O–Cu–O–Ca connectivity (Fig. 2 ▸). Such units are cross-linked by the μ_7_-BTC^3−^ anions to create a three-dimensional framework (Fig. 3 ▸). In addition, the terminal water mol­ecule is hydrogen bonded to the carboxyl­ate O atoms (Table 2 ▸), forming a two-dimensional network parallel to (100). π-π stacking inter­actions between (C1–C6) benzene rings [*Cg*⋯*Cg*(−*x*, 1 − *y*, 2 − *z*) = 3.357 (2) Å] further stabilize the crystal structure.

## Synthesis and crystallization   

The title compound was synthesized using a similar procedure to that for the synthesis of the analogous compound [CuSr_2_(BTC)_2_]·10H_2_O (Sun *et al.*, 2016 ▸). A mixture of H_3_BTC (210 mg, 1 mmol), CuCl_2_·6H_2_O (121 mg, 0.5 mmol) and CaCl_2_ (110 mg, 1 mmol) in 15 mL of distilled water was stirred for 10 min in air; 0.5 *M* NaOH was then added dropwise, and then the mixture was turned into a Parr Teflon-lined stainless steel vessel and heated to 443 K for 3 d. Blue block-shaped crystals suitable for X-ray diffraction were obtained in 60% yield (based on benzene-1,3,5-tri­carb­oxy­lic acid).

## Refinement   

Crystal data, data collection and structure refinement details are summarized in Table 3 ▸. The hydrogen atoms of the coordinating water mol­ecule were located from a difference-Fourier map, but refined using a riding model with isotropic displacement parameters *U*_iso_(H) = 1.2*U*_eq_(O). Hydrogen atoms attached to carbon atoms were positioned geometrically (C—H = 0.93 Å) and refined with *U*_iso_(H) = 1.2*U*_eq_(C).

## Supplementary Material

Crystal structure: contains datablock(s) I, global. DOI: 10.1107/S205698901700665X/xu5901sup1.cif

Structure factors: contains datablock(s) I. DOI: 10.1107/S205698901700665X/xu5901Isup2.hkl

CCDC reference: 1547715

Additional supporting information:  crystallographic information; 3D view; checkCIF report

## Figures and Tables

**Table 1:** Selected bond lengths (Å)

Ca1—O1	2.338 (2)	Ca1—O6^iv^	2.357 (2)
Ca1—O3^i^	2.280 (2)	Ca1—O*W*1	2.390 (2)
Ca1—O4^ii^	2.333 (2)	Cu1—O2	1.9435 (19)
Ca1—O5^iii^	2.466 (2)	Cu1—O5^v^	1.9800 (19)

Symmetry codes: (i) 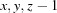; (ii) 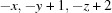; (iii) 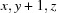; (iv) 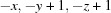; (v) 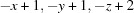.

**Table 2:** Hydrogen-bond geometry (Å, °)

*D*—H⋯*A*	*D*—H	H⋯*A*	*D*⋯*A*	*D*—H⋯*A*
O*W*1—H*W*1*A*⋯O4^v^	0.84 (1)	1.95 (1)	2.793 (3)	173 (3)
O*W*1—H*W*1*B*⋯O2^vi^	0.84 (1)	2.31 (2)	3.020 (3)	143 (3)

Symmetry codes: (v) 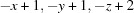; (vi) 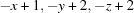.

**Table 3:** Experimental details

Crystal data	
Chemical formula	[Ca_2_Cu(C_9_H_3_O_6_)_2_(H_2_O)_2_]
*M*_r_	593.96
Crystal system, space group	Triclinic, *P* 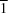
Temperature (K)	296
*a*, *b*, *c* (Å)	6.664 (3), 8.754 (4), 8.925 (4)
α, β, γ (°)	103.065 (4), 110.140 (4), 92.776 (5)
*V* (Å^3^)	471.6 (4)
*Z*	1
Radiation type	Mo *K*α
μ (mm^−1^)	1.79
Crystal size (mm)	0.18 × 0.15 × 0.14

Data collection	
Diffractometer	Bruker SMART CCD
Absorption correction	Multi-scan (*SADABS*; Bruker, 2009 ▸)
*T*_min_, *T*_max_	0.721, 0.766
No. of measured, independent and observed [*I* > 2σ(*I*)] reflections	2442, 1635, 1588
*R*_int_	0.012
(sin θ/λ)_max_ (Å^−1^)	0.595

Refinement	
*R*[*F* ^2^ > 2σ(*F* ^2^)], *wR*(*F* ^2^), *S*	0.029, 0.084, 1.04
No. of reflections	1635
No. of parameters	166
No. of restraints	3
H-atom treatment	H atoms treated by a mixture of independent and constrained refinement
Δρ_max_, Δρ_min_ (e Å^−3^)	0.35, −0.68

Computer programs: *APEX2* and *SAINT* (Bruker, 2009 ▸), *SHELXS97* and *SHELXTL* (Sheldrick, 2008 ▸), *SHELXL2014* (Sheldrick, 2015 ▸) and *DIAMOND* (Brandenburg, 2006 ▸).

## supplementary crystallographic information

### Crystal data

**Table:**

[Ca_2_Cu(C_9_H_3_O_6_)_2_(H_2_O)_2_]	*Z* = 1
*M**_r_* = 593.96	*F*(000) = 299
Triclinic, *P*1	*D*_x_ = 2.091 Mg m^−^^3^
Hall symbol: -P 1	Mo *K*α radiation, λ = 0.71073 Å
*a* = 6.664 (3) Å	Cell parameters from 1990 reflections
*b* = 8.754 (4) Å	θ = 2.4–27.5°
*c* = 8.925 (4) Å	µ = 1.79 mm^−^^1^
α = 103.065 (4)°	*T* = 296 K
β = 110.140 (4)°	Block, blue
γ = 92.776 (5)°	0.18 × 0.15 × 0.14 mm
*V* = 471.6 (4) Å^3^	

### Data collection

**Table:**

Bruker SMART CCD diffractometer	1635 independent reflections
Radiation source: fine-focus sealed tube	1588 reflections with *I* > 2σ(*I*)
Graphite monochromator	*R*_int_ = 0.012
φ and ω scans	θ_max_ = 25.0°, θ_min_ = 2.4°
Absorption correction: multi-scan (SADABS; Bruker, 2009)	*h* = −7→7
*T*_min_ = 0.721, *T*_max_ = 0.766	*k* = −10→4
2442 measured reflections	*l* = −10→10

### Refinement

**Table:**

Refinement on *F*^2^	Primary atom site location: structure-invariant direct methods
Least-squares matrix: full	Secondary atom site location: difference Fourier map
*R*[*F*^2^ > 2σ(*F*^2^)] = 0.029	Hydrogen site location: inferred from neighbouring sites
*wR*(*F*^2^) = 0.084	H atoms treated by a mixture of independent and constrained refinement
*S* = 1.04	*w* = 1/[σ^2^(*F*_o_^2^) + (0.0501*P*)^2^ + 0.6456*P*] where *P* = (*F*_o_^2^ + 2*F*_c_^2^)/3
1635 reflections	(Δ/σ)_max_ < 0.001
166 parameters	Δρ_max_ = 0.35 e Å^−^^3^
3 restraints	Δρ_min_ = −0.68 e Å^−^^3^

### Special details

**Table:**

Geometry. All esds (except the esd in the dihedral angle between two l.s. planes) are estimated using the full covariance matrix. The cell esds are taken into account individually in the estimation of esds in distances, angles and torsion angles; correlations between esds in cell parameters are only used when they are defined by crystal symmetry. An approximate (isotropic) treatment of cell esds is used for estimating esds involving l.s. planes.
Refinement. Refinement of F^2^ against ALL reflections. The weighted R-factor wR and goodness of fit S are based on F^2^, conventional R-factors R are based on F, with F set to zero for negative F^2^. The threshold expression of F^2^ > 2sigma(F^2^) is used only for calculating R-factors(gt) etc. and is not relevant to the choice of reflections for refinement. R-factors based on F^2^ are statistically about twice as large as those based on F, and R- factors based on ALL data will be even larger.

### Fractional atomic coordinates and isotropic or equivalent isotropic displacement parameters (Å^2^)

**Table:**

	*x*	*y*	*z*	*U*_iso_*/*U*_eq_	
Ca1	0.13337 (8)	0.79200 (6)	0.58531 (6)	0.01430 (16)	
Cu1	0.5000	1.0000	1.0000	0.01590 (16)	
O1	0.2949 (3)	0.6981 (2)	0.8180 (2)	0.0206 (4)	
O2	0.3877 (3)	0.8375 (2)	1.0782 (2)	0.0192 (4)	
O3	0.2155 (4)	0.5598 (2)	1.4590 (2)	0.0273 (5)	
O4	0.1993 (3)	0.2977 (2)	1.4102 (2)	0.0190 (4)	
O5	0.2085 (3)	0.0033 (2)	0.8405 (2)	0.0186 (4)	
O6	0.0233 (4)	0.1170 (2)	0.6527 (2)	0.0283 (5)	
C1	0.2707 (4)	0.5610 (3)	1.0114 (3)	0.0126 (5)	
C2	0.2808 (4)	0.5623 (3)	1.1690 (3)	0.0145 (5)	
H2A	0.3166	0.6577	1.2499	0.017*	
C3	0.2377 (4)	0.4211 (3)	1.2074 (3)	0.0136 (5)	
C4	0.1929 (4)	0.2786 (3)	1.0883 (3)	0.0142 (5)	
H4A	0.1703	0.1841	1.1148	0.017*	
C5	0.1815 (4)	0.2765 (3)	0.9288 (3)	0.0142 (5)	
C6	0.2163 (4)	0.4182 (3)	0.8895 (3)	0.0136 (5)	
H6A	0.2034	0.4176	0.7822	0.016*	
C7	0.3178 (4)	0.7085 (3)	0.9639 (3)	0.0139 (5)	
C8	0.2195 (4)	0.4278 (3)	1.3728 (3)	0.0154 (5)	
C9	0.1339 (4)	0.1255 (3)	0.7989 (3)	0.0142 (5)	
OW1	0.4854 (3)	0.8960 (3)	0.6111 (3)	0.0315 (5)	
HW1A	0.587 (4)	0.844 (3)	0.604 (4)	0.038*	
HW1B	0.544 (5)	0.9871 (18)	0.669 (4)	0.038*	

### Atomic displacement parameters (Å^2^)

**Table:**

	*U*^11^	*U*^22^	*U*^33^	*U*^12^	*U*^13^	*U*^23^
Ca1	0.0188 (3)	0.0142 (3)	0.0099 (3)	0.0011 (2)	0.0048 (2)	0.0040 (2)
Cu1	0.0204 (3)	0.0110 (2)	0.0147 (3)	0.00010 (17)	0.00383 (19)	0.00479 (18)
O1	0.0344 (11)	0.0158 (9)	0.0143 (9)	0.0058 (8)	0.0090 (8)	0.0084 (7)
O2	0.0282 (10)	0.0125 (9)	0.0145 (9)	−0.0024 (7)	0.0056 (8)	0.0035 (7)
O3	0.0480 (13)	0.0191 (10)	0.0181 (10)	0.0061 (9)	0.0181 (10)	0.0016 (8)
O4	0.0236 (10)	0.0189 (10)	0.0181 (9)	0.0014 (7)	0.0094 (8)	0.0092 (8)
O5	0.0228 (10)	0.0107 (9)	0.0180 (9)	0.0032 (7)	0.0027 (8)	0.0030 (7)
O6	0.0452 (13)	0.0206 (10)	0.0118 (10)	0.0027 (9)	0.0010 (9)	0.0051 (8)
C1	0.0118 (12)	0.0128 (12)	0.0131 (12)	0.0020 (9)	0.0033 (10)	0.0049 (10)
C2	0.0160 (12)	0.0128 (12)	0.0141 (12)	0.0015 (9)	0.0051 (10)	0.0032 (10)
C3	0.0148 (12)	0.0140 (12)	0.0124 (12)	0.0025 (9)	0.0049 (10)	0.0043 (10)
C4	0.0169 (12)	0.0119 (12)	0.0155 (12)	0.0035 (9)	0.0057 (10)	0.0065 (10)
C5	0.0143 (12)	0.0135 (12)	0.0131 (12)	0.0032 (10)	0.0030 (10)	0.0033 (10)
C6	0.0159 (12)	0.0130 (12)	0.0125 (12)	0.0044 (10)	0.0040 (10)	0.0057 (10)
C7	0.0145 (12)	0.0135 (12)	0.0167 (13)	0.0051 (9)	0.0063 (10)	0.0078 (10)
C8	0.0154 (12)	0.0174 (13)	0.0130 (12)	0.0024 (10)	0.0047 (10)	0.0036 (10)
C9	0.0177 (12)	0.0129 (12)	0.0129 (13)	0.0017 (10)	0.0056 (10)	0.0050 (10)
OW1	0.0262 (11)	0.0264 (11)	0.0459 (14)	0.0047 (9)	0.0166 (10)	0.0116 (10)

### Geometric parameters (Å, º)

**Table:**

Ca1—O1	2.338 (2)	O5—Ca1^viii^	2.466 (2)
Ca1—O3^i^	2.280 (2)	O6—C9	1.241 (3)
Ca1—O4^ii^	2.333 (2)	O6—Ca1^iv^	2.357 (2)
Ca1—O5^iii^	2.466 (2)	O6—Ca1^viii^	2.954 (2)
Ca1—O6^iv^	2.357 (2)	C1—C2	1.382 (4)
Ca1—OW1	2.390 (2)	C1—C6	1.395 (4)
Ca1—Cu1	3.6439 (13)	C1—C7	1.499 (3)
Cu1—O2^v^	1.9435 (19)	C2—C3	1.398 (4)
Cu1—O2	1.9435 (19)	C2—H2A	0.9300
Cu1—O5^vi^	1.9800 (19)	C3—C4	1.386 (4)
Cu1—O5^iii^	1.9800 (19)	C3—C8	1.511 (3)
Cu1—Ca1^v^	3.6439 (13)	C4—C5	1.395 (4)
O1—C7	1.239 (3)	C4—H4A	0.9300
O2—C7	1.278 (3)	C5—C6	1.393 (4)
O3—C8	1.242 (3)	C5—C9	1.485 (3)
O3—Ca1^vii^	2.280 (2)	C6—H6A	0.9300
O4—C8	1.271 (3)	C8—Ca1^ii^	3.141 (3)
O4—Ca1^ii^	2.333 (2)	C9—Ca1^viii^	3.104 (3)
O5—C9	1.278 (3)	OW1—HW1A	0.844 (10)
O5—Cu1^viii^	1.9800 (19)	OW1—HW1B	0.836 (10)
			
O3^i^—Ca1—O4^ii^	99.86 (8)	C7—O2—Cu1	110.48 (16)
O3^i^—Ca1—O1	81.17 (8)	C8—O3—Ca1^vii^	168.1 (2)
O4^ii^—Ca1—O1	87.46 (7)	C8—O4—Ca1^ii^	118.28 (16)
O3^i^—Ca1—O6^iv^	97.47 (8)	C9—O5—Cu1^viii^	125.84 (16)
O4^ii^—Ca1—O6^iv^	93.57 (8)	C9—O5—Ca1^viii^	107.73 (15)
O1—Ca1—O6^iv^	178.43 (7)	Cu1^viii^—O5—Ca1^viii^	109.61 (8)
O3^i^—Ca1—OW1	83.82 (8)	C9—O6—Ca1^iv^	157.26 (18)
O4^ii^—Ca1—OW1	173.99 (8)	C9—O6—Ca1^viii^	85.06 (15)
O1—Ca1—OW1	88.42 (8)	Ca1^iv^—O6—Ca1^viii^	114.30 (8)
O6^iv^—Ca1—OW1	90.64 (8)	C2—C1—C6	120.0 (2)
O3^i^—Ca1—O5^iii^	148.03 (7)	C2—C1—C7	122.6 (2)
O4^ii^—Ca1—O5^iii^	91.33 (7)	C6—C1—C7	117.4 (2)
O1—Ca1—O5^iii^	69.43 (7)	C1—C2—C3	120.3 (2)
O6^iv^—Ca1—O5^iii^	111.71 (7)	C1—C2—H2A	119.8
OW1—Ca1—O5^iii^	83.12 (7)	C3—C2—H2A	119.8
O3^i^—Ca1—Cu1	118.21 (6)	C4—C3—C2	119.6 (2)
O4^ii^—Ca1—Cu1	110.50 (5)	C4—C3—C8	120.9 (2)
O1—Ca1—Cu1	49.46 (5)	C2—C3—C8	119.2 (2)
O6^iv^—Ca1—Cu1	131.04 (6)	C3—C4—C5	120.3 (2)
OW1—Ca1—Cu1	63.49 (6)	C3—C4—H4A	119.9
O5^iii^—Ca1—Cu1	30.79 (4)	C5—C4—H4A	119.9
O6^iii^—Ca1—Cu1	73.03 (4)	C6—C5—C4	119.8 (2)
C9^iii^—Ca1—Cu1	50.47 (5)	C6—C5—C9	118.8 (2)
C8^ii^—Ca1—Cu1	106.72 (6)	C4—C5—C9	121.4 (2)
O2^v^—Cu1—O2	180.000 (1)	C5—C6—C1	119.9 (2)
O2^v^—Cu1—O5^vi^	91.13 (8)	C5—C6—H6A	120.1
O2—Cu1—O5^vi^	88.87 (8)	C1—C6—H6A	120.1
O2^v^—Cu1—O5^iii^	88.87 (8)	O1—C7—O2	123.6 (2)
O2—Cu1—O5^iii^	91.13 (8)	O1—C7—C1	118.5 (2)
O5^vi^—Cu1—O5^iii^	180.000 (1)	O2—C7—C1	117.8 (2)
O2^v^—Cu1—Ca1^v^	87.31 (6)	O3—C8—O4	124.8 (2)
O2—Cu1—Ca1^v^	92.69 (6)	O3—C8—C3	117.4 (2)
O5^vi^—Cu1—Ca1^v^	39.61 (5)	O4—C8—C3	117.7 (2)
O5^iii^—Cu1—Ca1^v^	140.39 (5)	O6—C9—O5	120.3 (2)
O2^v^—Cu1—Ca1	92.69 (6)	O6—C9—C5	121.0 (2)
O2—Cu1—Ca1	87.31 (6)	O5—C9—C5	118.7 (2)
O5^vi^—Cu1—Ca1	140.39 (5)	Ca1—OW1—HW1A	127 (2)
O5^iii^—Cu1—Ca1	39.61 (5)	Ca1—OW1—HW1B	122 (2)
Ca1^v^—Cu1—Ca1	180.0	HW1A—OW1—HW1B	105.9 (16)
C7—O1—Ca1	146.05 (17)		

Symmetry codes: (i) *x*, *y*, *z*−1; (ii) −*x*, −*y*+1, −*z*+2; (iii) *x*, *y*+1, *z*; (iv) −*x*, −*y*+1, −*z*+1; (v) −*x*+1, −*y*+2, −*z*+2; (vi) −*x*+1, −*y*+1, −*z*+2; (vii) *x*, *y*, *z*+1; (viii) *x*, *y*−1, *z*.

### Hydrogen-bond geometry (Å, º)

**Table:**

*D*—H···*A*	*D*—H	H···*A*	*D*···*A*	*D*—H···*A*
O*W*1—H*W*1*A*···O4^vi^	0.84 (1)	1.95 (1)	2.793 (3)	173 (3)
O*W*1—H*W*1*B*···O2^v^	0.84 (1)	2.31 (2)	3.020 (3)	143 (3)

Symmetry codes: (v) −*x*+1, −*y*+2, −*z*+2; (vi) −*x*+1, −*y*+1, −*z*+2.
